# Role of social support in the relationship between resilience and sleep quality among cancer patients

**DOI:** 10.3389/fpsyt.2024.1310118

**Published:** 2024-04-16

**Authors:** ChunYing Cui, Lie Wang

**Affiliations:** ^1^School of Humanities and Management, Wannan Medical College, Wuhu, Anhui, China; ^2^School of Public Health, China Medical University, Shenyang North New Area, Shenyang, Liaoning, China

**Keywords:** sleep quality, social support, resilience, cancer, mediating and moderating effect

## Abstract

**Introduction:**

The present study aimed to investigate the effect of resilience on sleep quality and explore the role of social support between resilience and sleep quality in cancer patients.

**Methods:**

A multicenter and cross-sectional study was conducted in China from May to November 2021. A total of 202 cancer patients were recruited to complete the questionnaires composed of demographic information, Pittsburg Sleep Quality Index (PSQI), Resilience Scale-14 (RS-14), and Multidimensions Scale of Perceived Social Support (MSPSS). The associations between resilience, social support, and sleep quality were explored through hierarchical regression analysis.

**Results:**

The prevalence of poor sleep quality was 50% among cancer patients. Resilience, social support, and the interaction between resilience and social support were all found to be significantly associated with sleep quality. Results of simple slope analysis indicated that the association between resilience and sleep quality were gradually decreased with the increasing social support levels (1 SD below the mean, *B*=-0.225, *β*=-0.551, *P*<0.001), mean social support (*B*=-0.147, *β*=-0.353, *P*<0.001) and high social support (1 SD above the mean, *B*=-0.065, *β*=-0.156, *P*<0.001). Additionally, social support mediated the effect of resilience on sleep quality among cancer patients.

**Discussion:**

Poor sleep quality has been common in cancer patients. Social support could mediate and alleviate the relationship between resilience and sleep quality among cancer patients. Besides providing sufficient social support, interventions based on resilience should be applied to address sleep problems in cancer patients.

## 1 Introduction

Sleep disorders have become a frequent concern in chronic disease patients, particularly cancer patients. Sleep disorders constitute a clinical syndrome characterized by disruptions in the sleep-wake cycle, resulting in abnormal sleep behaviors and decreased sleep quality. An emerging body of evidence reported that sleep disturbance has been regarded as the most burdensome symptom (1, 2). Furthermore, 30%-93.1% of cancer patients (3, 4) had sleep disorders, and were significantly higher than 11%-32% general population (5, 6). Sleep disorders were related to decrease quality of life and energy, and increase psychological and physical problems in patients with cancer (7). More importantly, sleep deprivation negatively regulates immunological and inflammatory functions, which may change cognition, memory, and emotional instability and decrease appetite (8). In addition, sleep problems in cancer patients negatively affected their family caregivers’ sleep quality (9).

However, compared to psychological distress and well-being, sleep problems in cancer patients get less empirical attention. Furthermore, most studies focus on the effect of negative factors on sleep problems. For example, Papadopoulos et al. (10) found that anxiety and stress were independent correlates of sleep quality after adjusting confounding variables in lung cancer patients. Besides, He et al. indicated that psychological fear of cancer and medicine therapy could be significant reasons to explain sleep disorders among patients with thyroid cancer (11). In turn, severe sleep problems could deteriorate these mental symptoms (12). However, protective factors of sleep quality are neglected in cancer patients, such as positive psychological resources.

Resilience, as a positive psychological resource, has gotten increasing attention in the oncology psychology field and is defined as a developmental capability that an individual can bounce back or rebound from failure, tragedy, frustration, and other adverse events (13). Furthermore, high resilience helps individuals cope with disease diagnosis, treatment, and prognosis and improve health-related quality of life (14–17). Moreover, a meta-analysis (18) showed that sleep quality and resilience are positively correlated amongst non-clinical and healthy populations. Studies found that cancer patients with similar diseases and treatment status had significantly different quality of life, possibly due to variations in resilience (19, 20). Therefore, resilience could help explain the variation in sleep quality among cancer patients. However, to our best knowledge, few studies explored the positive role of resilience on sleep quality in patients with cancer.

Social support refers to a combination of perceiving assistance available from family, friends, and other significant relationships in times of need (21). Specifically, it is a multidimensional concept including emotional, informational, and instrumental aspects. Many researchers reported that sufficient social support could relieve psychological distress and enhance disease adaptation in various cancer patients (22–24). Based on the theory of the “stress-buffering hypothesis” (25), social support can enhance individuals’ well-being and health by acting as a “direct agent.” Besides, it can act as an “antecedent factor” to increase individuals’ positive coping styles and psychological empowerment, which thus has a positive effect on mental health and quality of life. For instance, Ng et al. (26) suggested that social support helped directly reduce sleep disorders and improve sleep quality in Hemodialysis Patients. In addition, van Schalkwijk et al. (27) indicated that social support could moderate the negative effect of stress on sleep among adolescents. The association between social support and sleep quality has been explored in older men (28), adolescents (27), and patients with chronic diseases (26). Therefore, our study hypothesized that social support influenced the associations between resilience and sleep disorders in cancer patients.

Given the above concerns, our study hypothesized that the association between resilience and sleep quality is weak for the cancer patients with high level of social support. Additionally, resilience could indirectly influence sleep quality by social support among cancer patients. Therefore, the present study aimed to investigate the effect of resilience on sleep quality and explore the role of social support between resilience and sleep quality in cancer patients.

## 2 Materials and methods

### 2.1 Study design and sample

This study, a multicenter and cross-sectional design, was conducted in Shenyang, Liaoning province, China, from May to November 2021. Participants were recruited using convenience sampling the Department of Oncology at Liaoning Provincial People’s Hospital, Hunnan International Hospital-the first hospital of China Medical University, the First Affiliated Hospital of China Medical University and the Affiliated Shengjing Hospital of China Medical University. The inclusion criteria of this study included that patient: 1) were diagnosed with cancer; 2) were able to understand and communicate with Chinese; 3) were at least 18 years old. The exclusion criteria of this study included that patient: 1) had other severe chronic diseases, including heart failure, chronic obstructive pulmonary disease, asthma, chronic pulmonary heart disease, chronic respiratory failure; 2) had history of psychiatry disorders; 3) had cognitive and intellectual disorders. Each eligible patient would be given self-reported questionnaires after providing written informed consent for this study. Finally, 202 patients completed the survey out of 230 cancer patients, resulting in an effective response rate of 87.8%. Twenty patients declined to participate in the investigation, and five participants were excluded due to invalid data (missing data >30%). Additionally, three patients with COPD, chronic pulmonary heart disease, and heart failure, respectively, were excluded. Therefore, our study included 202 cancer patients. The procedures used in our survey were approved by the Committee on Human Experimentation of the First Affiliated Hospital of China Medical University (NO. 2021-430-2).

### 2.2 Measurement of sleep quality

The present study adopted the Chinese version of the Pittsburg Sleep Quality Index (PSQI) (29) to evaluate the sleep quality or sleep problems of patients with cancer in the last month. This scale has seven components: subjective sleep quality, daytime dysfunction, sleep latency, use of medication for sleep, sleep duration, sleep efficiency, and sleep disorders. It comprises 19 items, and the global score ranges from 0 to 21. A score of >5 is regarded as a cut-off value, indicating poor sleep quality in cancer patients. A higher score on the PSQI indicates more severe sleep problems (30). The Cronbach’s coefficient for the PSQI was 0.778 in this study.

### 2.3 Measurement of resilience

The level of resilience was measured using the Chinese version of the Resilience Scale-14 (RS-14) (31) among cancer patients in the present study. Each item of RS-14 adopts a 7-point Likert type scale ranging from “1=very strongly disagree” to “7=very strongly agree”. The summed score of RS-14 ranges from 14 to 98, with higher scores indicating a higher level of resilience. The Cronbach’s coefficient for the RS-14 was 0.884 in the present study.

### 2.4 Measurement of social support

The Chinese version of the Multidimensions Scale of Perceived Social Support (MSPSS) (32) was used to assess the level of social support in cancer patients. The scale includes 12 items that are scored on a 7-point Likert type (from 1= “very strongly disagree” to 7= “very strongly agree”) and comprises three dimensions: family support, friend support, and significant other support. The total score is from 12 to 84, with a higher score indicating more social support among cancer patients. In the present study, the Cronbach’s coefficient for the MSPSS was 0.874.

### 2.5 Demographic information

In addition, several demographic variables were included in the present study, including age, gender (male and female), Body Mass Index (BMI, <18.5, 18.5-23.9, 24-27.9 and ≥28), marital status (single/separation/widowed/divorced and married/cohabited), educational background (junior school and below, high school, junior college and bachelor degree or above), employment status (unemployment and part-time/full-time) and monthly income (≤3,000, ≤5,000 and >5,000).

### 2.6 Statistical analysis

T-tests and one-way ANOVAs were used to examine group differences in sleep quality. The correlation coefficient (*r*) in continuous variables was tested by using correlation analysis. The associations of social support and resilience with sleep quality and the moderating effect of resilience were explored by adopting hierarchical regression analysis. Specifically, besides age and gender, demographic variables associated with sleep quality in univariate analysis (*P*<0.25) were adjusted and entered in step 1. Resilience was added in step 2. Then, in step 3, social support was entered. Finally, the interaction of resilience*social support was entered. There was a moderating role of social support between resilience and sleep quality if the interaction was significant (*P*<0.05) in regression analysis. Furthermore, our study adopted simple slope analysis to visualize the interaction term. Before regression analysis, these variables were centralized. Asymptotic and resampling strategies examined the mediating role of social support. The bootstrap estimate was based on 5000 bootstrap samples. The bias-corrected and accelerated 95% confidence interval (BCa 95% CI) for mediation was calculated, and a BCa 95% CI excluding 0 indicated a significant mediating role. SPSS 20.0 software was used for statistical analysis, and the significance level was *P*-value <0.05.

## 3 Results

### 3.1 Descriptive statistics

**Table 1** presents demographic information and group differences in sleep quality among cancer patients. Among these patients, more than half were above 55 years old, and 50.5% (102) subjects were male. Among the patients, 82.7% (167) reported being married or cohabited, 54.0% (109) had education levels of junior school or below, and 51.0% (103) had a monthly income level of ≤ 3,000 yuan (CNY). The main cancer diagnoses included lung cancer (15.3%), breast cancer (14.4%), bone cancer 8.9%), head and neck cancer (7.4%), among others. In summary, 89.1% (180) of subjects were unemployed, and they reported poorer sleep quality compared to patients who were part-time or full-time (*P*<0.029).

**Table 1:** Distribution of demographic data and the results of univariate analysis.

Variables	N (*%*)	Sleep quality -PSQI score	*t/F*	*P*
		Mean ± SD		
Age			1.659	0.099
≤ 55	81(40.1)	6.02 ± 4.06		
> 55	121(59.9)	7.01 ± 4.18		
Gender			0.827	0.410
Male	100(49.5)	6.37 ± 3.84		
Female	102(50.5)	6.85 ± 4.43		
BMI			0.479	0.698
<18.5	16(7.9)	7.44 ± 4.80		
18.5-23.9	114(56.4)	6.62 ± 4.00		
24-27.9	55(27.2)	6.64 ± 4.21		
≥ 28	17(8.4)	5.71 ± 4.48		
Marital status			0.693	0.489
Single/separation/widowed/divorced	35(17.3)	6.17 ± 4.13		
Married/cohabited	167(82.7)	6.71 ± 4.16		
Educational background			1.757	0.157
Junior school and below	109(54.0)	6.90 ± 3.95		
High school	45(22.3)	7.11 ± 4.92		
Junior college	22(10.9)	6.00 ± 4.34		
Bachelor degree or above	26(12.9)	5.07 ± 2.92		
Employment status			2.289	0.029
Unemployment	180(89.1)	6.81 ± 4.19		
Part-time/full-time	22(10.9)	5.00 ± 3.41		
Monthly income			2.239	0.109
≤ 3,000	103(51.0)	7.19 ± 4.34		
≤ 5,000	52(25.7)	5.79 ± 3.77		
> 5,000	47(23.3)	6.26 ± 4.01		
Cancer type			1.152	0.318
Lung cancer	31(15.5)	7.19 ± 4.37		
Breast cancer	29(14.4)	5.41 ± 3.45		
Bone cancer	18(8.9)	7.28 ± 4.18		
Head and neck cancer	15(7.4)	5.13 ± 3.78		
Colorectal cancer	12(5.9)	5.17 ± 3.30		
Cervical cancer	11(5.4)	6.82 ± 4.79		
Esophageal cancer	10(5.0)	6.40 ± 4.09		
Liver cancer	4(2.0)	7.75 ± 3.95		
Endometrial cancer	3(1.5)	5.33 ± 2.52		
Vaginal cancer	3(1.5)	9.0 ± 5.29		
Gastric cancer	2(1.0)	7.50 ± 2.12		
Ovarian cancer	2(1.0)	12.5 ± 2.12		
Other type	9(4.5)	8.67 ± 6.46		
Miss	53(26.2)	6.70 ± 6.46		

### 3.2 Correlations among continuous variables

The results of correlation analysis for continuous variables are presented in **Table 2**. Age was found to have a positive correlation with sleep quality, as indicated by PSQI score (*r*=0.169, *P*<0.05). Additionally, both resilience (*r*=-0.443, *P*<0.01) and social support (*r*=-0.431, *P*<0.01) were negatively correlated with sleep quality, as measured by the PSQI score.

**Table 2:** Correlations among continues variables.

Variable	Mean	SD	Age	BMI	Resilience	Social support
Age	55.83	14.94	1			
BMI	22.97	3.42	0.032	1		
Resilience	70.88	9.97	-0.009	0.158^*^	1	
Social support	63.12	8.21	-0.074	0.142^*^	0.283^**^	1
Sleep quality -PSQI score	6.61	4.15	0.169^*^	-0.071	-0.443^**^	-0.431^**^

BMI, Body Mass Index; PSQI, Pittsburg Sleep Quality Index. SD, Standard Deviations.

*, P<0.05; **, P<0.01.

### 3.3 Hierarchical regression analysis

**Figure 1:**
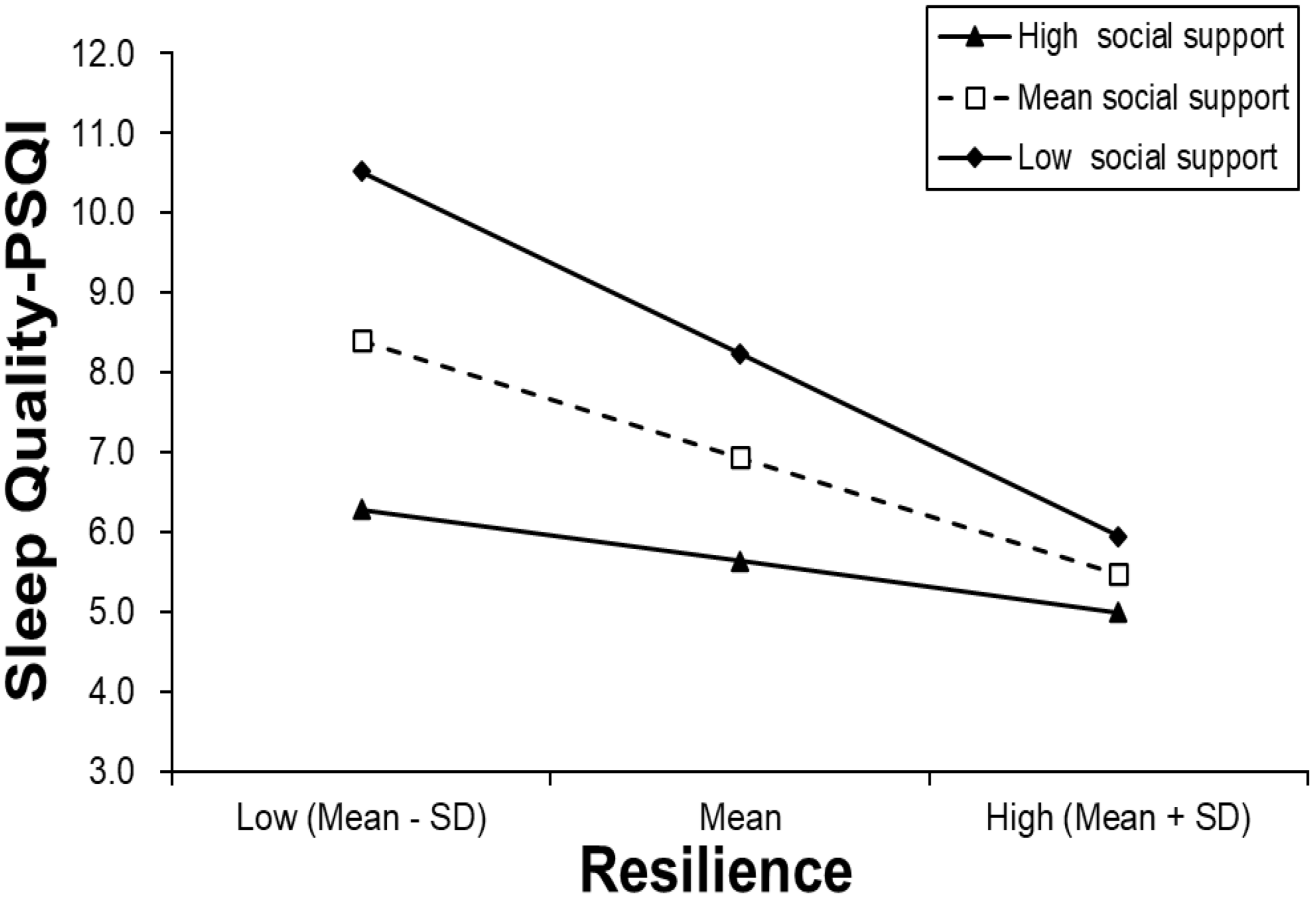
Simple slope plot of interaction between resilience and social support on sleep quality-PSQI score.

The results of the regression analysis for sleep quality are presented in **Table 3**. In step 1, demographic information, including age, gender, educational background, employment, and monthly income, was found to be significantly associated with sleep quality-PSQI score (*F*=2.309, adjusted *R*^2^ = 0.032, *P*<0.01). In step 2, resilience (b=-0.443, *P*<0.001) was found to be negatively related to sleep quality-PSQI score (*F*=10.463, adjusted *R*^2^ = 0.220, *R*^2^-change=0.118, *P*<0.001). In step 3, both resilience (b=-0.344, *P*<0.001) and social support (b=-0.323, *P*<0.001) were found to be significantly associated with sleep quality-PSQI score, and they improved the model fit (*F*=14.057, adjusted *R*^2^ = 0.313, *R*^2^-change=0.281, *P*<0.001). In step 4, the interaction of resilience*social support was found to be significantly and positively associated with sleep quality-PSQI score (b=0.207, *P*<0.001) and explained an additional 4.1% of the variance for sleep quality (*F*=14.660, adjusted *R*^2^ = 0.352, *R*^2^-change=0.041, *P*<0.001). In addition, the results of simple slope analysis indicated that the association of resilience with sleep quality was gradually decreased with an increase in social support level: for low social support (1 SD below the mean, B=-0.225, b=-0.551, *P*<0.001), mean social support (B=-0.147, b=-0.353, *P*<0.001) and high social support (1 SD above the mean, B=-0.065, b=-0.156, *P*<0.001). The interaction is visualized in **Figure 1**.

**Table 3:** The hierarchical multiple linear regression analysis results of sleep quality - PSQI score.

Variables	*B*	*SE B*	*β*	*T*	*P*-value
Block 1					
Age	0.043	0.021	0.156	2.077	0.039
Gender	0.670	0.582	0.081	1.151	0.251
Educational background	-0.163	0.331	-0.042	0.494	0.622
Employment	-0.937	1.006	-0.071	0.931	0.353
Monthly income	-0.404	0.424	-0.080	0.953	0.342
* F*	2.309				<0.01
Adjusted *R*^2^	0.032				
Block 2					
Resilience	-0.184	0.026	-0.443	6.960	<0.001
* F*	10.463				<0.001
Adjusted *R*^2^	0.220				
* R*^2^-change	0.188				<0.001
Block 3					
Resilience	-0.143	0.026	-0.344	5.493	<0.001
Social support	-0.163	0.031	-0.323	5.214	<0.001
* F*	14.057				<0.001
Adjusted *R*^2^	0.313				
* R*^2^-change	0.281				<0.001
Block 4					
Interaction	0.010	0.003	0.207	3.587	<0.001
* F*	14.660				<0.001
Adjusted *R*^2^	0.352				
* R*^2^-change	0.041				<0.001

Additionally, the results of Asymptotic and resampling strategies revealed a partial mediating role of social support in the relationship between resilience and sleep quality among cancer patients. The effect size was -0.986 with BCa 95% confidence interval of -0.1706 to -0.0419, indicating that the proportion of social support mediating effect for resilience was 22.3%.

## 4 Discussion

The present study examined the association between social support and sleep quality and explored its moderator in Chinese cancer patients. Our results indicated that 50.0% of participants reported poor sleep quality (PSQI score >5), a higher percentage than that reported by cancer survivors in the USA nine years post-diagnosis A (PSQI score >5: 19.5%) (33). Furthermore, the prevalence of poor sleep quality in our study was significantly higher than that observed in other studies of advanced cancer patients (PSQI score >5: 40%) (34). The high prevalence in our study can be attributed to the fact that it was conducted during COVID-19 pandemic. Cancer patients may encounter additional stressors during the pandemic, such as treatment delays, increased risk of infection, and limited access to follow-up care, all of which could adversely affect their sleep quality (35–37). Additionally, some studies have reported that changes in sleep habits due to lockdown measures could lead to a decrease in sleep quality and even cause insomnia (38, 39). Its high prevalence of sleep disturbances among cancer patients underscores the importance of identifying correlates of sleep quality and designing targeted interventions for this population.

Based on the results, our study suggests that resilience has a direct effect on sleep quality and indirect effect mediated by social support among cancer patients. The direct association of resilience with sleep quality in our study aligns with findings from previous research (40). The result show that resilience is crucial for cancer patients’ sleep quality. High-resilience individuals are better equipped to handle failures, uncertainty, and conflicts (41). Resilient individuals are adept at addressing adverse events and adapting to significant life changes, such as diseases, as they emerge from challenges stronger, more powerful, and wiser (42). Furthermore, Zhang and colleagues found that resilience was associated with quality of life in patients with breast cancer (43). In addition, several studies have reported that enhancing resilience significantly reduces mental health problems (44, 45). Resilience serves as an effective coping resource against adverse outcomes associated with cancer and its treatment, such as psychological distress, low quality of life and poor sleep quality.

Moreover, the mediating role of social support between resilience and sleep quality in the present study suggests that patients with higher levels of resilience are inclined to receive increased social support, thereby improving the sleep quality of cancer patients. Social support enables cancer patients to attend appointments, undergo diagnostic tests and procedures, and receive emotional sustenance during cancer therapy. Furthermore, Shahrokni et al. (46) found that older cancer patients may rely on more social support to cope with adverse events during cancer treatment. As a result, the effective effects of social support could mitigate sleep problems and enhance the level of sleep quality among patients with cancer. An emerging study (47) indicated that social support could alleviate depression and anxiety by providing social and emotional support as well as empathy from family, friends, and other significant groups. Subsequently, the combination of negative emotions could impact sleep quality, as individuals with anxiety and depression often experienced difficulties in falling asleep, which underscored the indirect relationship between social support and sleep quality. Give that our study was conducted during COVID-19 pandemic, lockdowns and gathering restrictions may have resulted in reduced social support from family, friends, and patient support groups for many cancer patients, thereby further decreasing their sleep quality.

To our knowledge, this study is the first to investigate the moderating role of social support in the relationship between resilience and sleep quality among cancer patients. The result of the simple slope analysis indicated that social support could attenuate the association between resilience and sleep quality, which suggested that cancer patients with higher levels of social support may maintain better sleep quality, even with lower levels of reliance, as they have access to other positive psychological resources, such as self-efficacy, hope, and optimism. Therefore, enhancing social support may be beneficial in addressing cancer-related sleep problems.

Several clinical implications should be emphasized in practice. It is crucial to take cancer-related outcomes seriously, particularly addressing sleep disturbance and psychological distress during diagnosis, treatment, and prognosis. Sleep disturbance is not only associated with an increased risk of cardiovascular diseases and infection (48), but it is also lined to decreased cognitive function (49) and heightened fatigue (50). The current approaches to treating sleep disorders in cancer patients primarily center around stress management training and medicine use. Our study identified another avenue for addressing and understanding sleep disturbances in cancer patients by providing social support, including communication exercises aimed at improving the expression of emotions and experiences related to the disease. Resilience increases programs also appear to be effective in improving sleep quality among cancer patients.

According to mindfulness-based cognitive therapy and acceptance and commitment therapy, increasing resilience has been shown to be effective in addressing sleep problems among patients with cancer (51). Furthermore, cancer patients could benefit from training in cognitive flexibility and discussions about changes in values and basic attitudes since the onset of the disease to enhance resilience (52).

There were some limitations in this study. Firstly, a cross-sectional survey design was employed, which posed challenges in establishing casual associations between resilience and social support with sleep quality among cancer patients. The mediation effect analysis using longitudinal data can effectively mitigate estimation bias issues inherent in cross-sectional research, providing robust evidence for elucidating the causal direction and mediation mechanism between variables, and has emerged as a new focal point in mediation research (53). Hence, longitudinal designs should be employed in future studies to replicate our findings. Secondly, resilience, social support, and sleep quality were assessed by adopting self-reported scales, which might result in recall and response bias. Currently, there is a growing use of objective measurements of sleep. To obtain more accurate results, future studies should consider adopting composite assessments with a combination of objective and subject methods. For instance, sleep assessment can utilize a combination of patient-reported outcome methods and objective methods, including polysomnography or actigraphy (34). Thirdly, while our study explored the relationships between resilience, social support, and sleep quality, other important clinical information was not collected in this study, including cancer stage and treatment methods. Hence, the effect of these possible risk factors on sleep quality should be explored in further research. Fourthly, all cancer patients included in the present study were from China, which may limit the generalizability of the findings. Diverse cultural settings should be considered in future research to enhance the applicability of the results. Finally, it is important to note that our study had a limited sample size, comprising only 202 cancer patients recruited through convenience sampling. The small sample size and sampling method may affect representativeness of our findings. Thus, future research should prioritize recruiting larger and more diverse samples to enhance the generalizability of the results.

## Conclusion

In summary, our findings indicated that poor sleep quality have been common among cancer patients, with 50% patients reporting sleep problems such as low sleep efficiency, sleep medicine use, and long sleep latency. Resilience and social support were found to be significantly and positively associated with sleep quality. Moreover, social support was found to alleviate and mediate the relationship between resilience and sleep quality among cancer patients. Besides ensuring sufficient social support, interventions based on resilience should be implemented as preventive and therapeutic methods for sleep problems in this population.

## Data availability statement

The raw data supporting the conclusions of this article will be made available by the authors, without undue reservation.

## Ethics statement

The studies involving humans were approved by the Committee on Human Experimentation of the First Affiliated Hospital of China Medical University (NO. 2021-430-2) The studies were conducted in accordance with the local legislation and institutional requirements. The participants provided their written informed consent to participate in this study.

## Author contributions

CC: Conceptualization, Data curation, Formal Analysis, Investigation, Methodology, Writing – original draft, Writing – review & editing. LW: Conceptualization, Data curation, Investigation, Writing – review & editing.
